# Follow-Up SARS-CoV-2 PCR Testing Outcomes From a Large Reference Lab in the US

**DOI:** 10.3389/fpubh.2021.679012

**Published:** 2021-05-31

**Authors:** Adam Sullivan, David Alfego, Brian Poirier, Jonathan Williams, Dorothy Adcock, Stan Letovsky

**Affiliations:** Labcorp^®^ Holdings of America, Inc., Burlington, NC, United States

**Keywords:** COVID-19, laboratory, repeat infection, PCR, real world data

## Abstract

By analyzing COVID-19 sequential COVID-19 test results of patients across the United States, we herein attempt to quantify some of the observations we've made around long-term infection (and false-positive rates), as well as provide observations on the uncertainty of sampling variability and other dynamics of COVID-19 infection in the United States. Retrospective cohort study of a registry of RT-PCR testing results for all patients tested at any of the reference labs operated by Labcorp^®^ including both positive, negative, and inconclusive results, from March 1, 2020 to January 28, 2021, including patients from all 50 states and outlying US territories. The study included 22 million patients with RT-PCR qualitative test results for SARS-CoV-2, of which 3.9 million had more than one test at Labcorp. We observed a minuscule <0.1% basal positive rate for follow up tests >115 days, which could account for false positives, long-haulers, and/or reinfection but is indistinguishable in the data. In observing repeat-testing, for patients who have a second test after a first RT-PCR, 30% across the cohort tested negative on the second test. For patients who test positive first and subsequently negative within 96 h (40% of positive test results), 18% of tests will subsequently test positive within another 96-h span. For those who first test negative and then positive within 96 h (2.3% of negative tests), 56% will test negative after a third and subsequent 96-h period. The sudden changes in RT-PCR test results for SARS-CoV-2 from this large cohort study suggest that negative test results during active infection or exposure can change rapidly within just days or hours. We also demonstrate that there does not appear to be a basal false positive rate among patients who test positive >115 days after their first RT-PCR positive test while failing to observe any evidence of widespread reinfection.

## Introduction

Examining the repeat-testing results of reverse transcription polymerase chain reaction (RT-PCR) testing for COVID-19 via nasal, nasopharyngeal, or oropharyngeal swab can help to derive population-level testing sensitivity and specificity levels as well as search for evidence of various logistical or operational false positives and negatives that could be systemic in nature (1). Despite strict guidelines for EUA approval of various RT-PCR testing platforms, population-level and epidemiological-level analysis shows that RT-PCR can have varying degrees of population-level sensitivity and specificity (2–5). For example, in looking at the sensitivity and specificity of PCR testing in the literature survey, a study out of Singapore showed that the combination of RT-PCR and chest tomography (CT) has higher sensitivity (91.9%,79/86) than RT-PCR alone (78.2%, 68/87), CT alone (66.7%, 54 of 81) or combination of two RT-PCR tests (86.2%, 75/87) (6). It is reported that population-level false negative rates can be as low as 3% and as high as 33% (7).

In this study, we use repeat-testing outcomes to attempt to quantify some of the population-level false positive and false negative rates for various time points. To use repeat- testing outcomes to infer single test sensitivity/specificity, we examined published literature on repeat testing outcomes. In one study examining repeat testing of head/neck injury surgeries in acute care settings, 43 of 52 patients required two or more preoperative PCR tests. Four (9.3%) had discrepant results (positive/negative) (8). In the case of an 81-year-old female who underwent urgent coronary artery bypass grafting and was readmitted following discharge to a nursing facility with a cluster of COVID-19 cases, despite symptomatology and imaging concerns for COVID-19, her two initial RT-PCR tests were negative, but a third test was positive (9).

Related to looking at negative PCR tests, a retrospective analysis was performed early in the pandemic by the French government, who decided to repatriate the 337 French nationals living in Wuhan and place them in quarantine in their home country (not a Labcorp tested site). They were all tested for SARS-CoV-2 twice and all tests at day 0 and day 5 were negative for RT-PCR testing (10). In another study, it was reported that 610 hospitalized patients clinically diagnosed with SARS-CoV-2 suffered from false negatives and multiple testing outcomes for repeat testing of the same patient (11). In a preprint systematic review of five studies involving 957 patients (“under suspicion of Covid-19” or with “confirmed cases”), false negatives ranged from 2 to 29%, although the researchers raise concerns of the lack of blinding to index-test results, failure to report key RT-PCR characteristics, among others (12).

Not specific to Labcorp's testing, there are also various investigations into the methods by which false positives and false negatives can occur. Various studies of optimal methods have demonstrated the nasopharyngeal (NP) swab to capture up to twice as many positive tests as the oropharyngeal (OP) swab, as confirmed in Tang et al. (13). Additionally, it was reported there are various operational false positives that are possible, including swab cross-contamination, contamination of reagents, or cross-reactions, where the operational false-positive rate was estimated to be 0.8 and 4% (14).

Using Labcorp's COVID-19 registry (2) allowed us to investigate follow-up PCR testing at a participating laboratory after an initial NP, at-home nasal collection specimens, or OP swab with the goal of identifying rates of positivity over time on a population-level analysis. The studies above are from patients often from non-US countries with unknown EUA operational guidelines, and test manufacturers outside the United States. An additional aim of this study is to shed light on the basal false positive rates in testing in the United States as well as understand the dynamics of a negative result in the context of an epidemiological study.

## Materials and Methods

The registry maintained by Labcorp includes 29.8 million (and counting) SARS-CoV-2 PCR tests conducted through multiple channels, including physician ordered, drive-through testing sites, employer/government contracts, and Pixel by Labcorp (Labcorp's at-home PCR test offering). Patients include a wide representation of the United States and specimen collections during the entire pandemic duration. Tests used in this analysis reflect all those who had repeat testing from March 1, 2020 to January 28, 2021 and reflect those who had at least one PCR test following an initial test, considered the patient's index. Analysis was conducted using Python™ 3.6.

## Results

**Figure 1:**
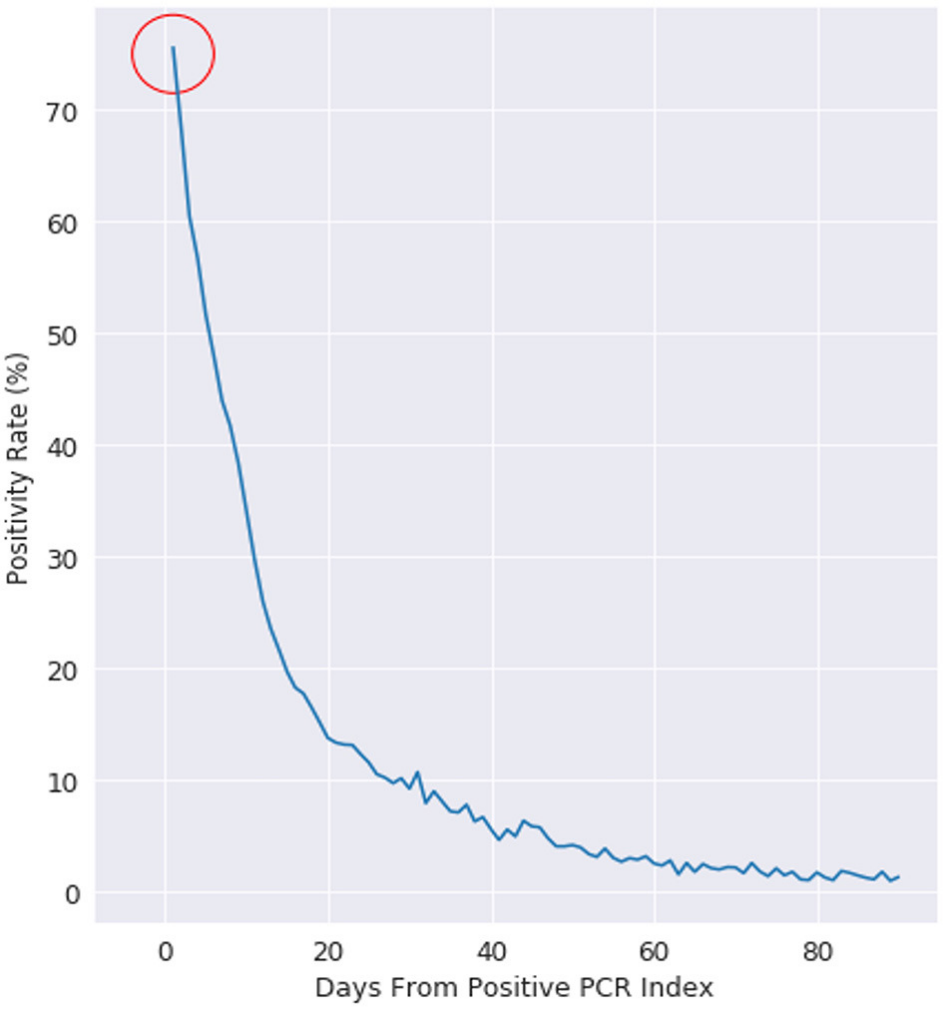
Positivity rate of PCR positive patients, where day 0 is the day of a patients first positive test. This figure is plotted for all US patients with more than 1 PCR test after a positive PCR test.

We first consider that every PCR test can have multiple outcomes: positive, negative, or other (where other can include collection issues, contamination, damage, mislabeling, or other logistical issues with sample collection). Every PCR positive patient is labeled with an index date of when they received their first positive PCR test. Figure 1 shows that for patients who receive their first positive PCR test with Labcorp and have a subsequent PCR test collected *the next day*, 25% of those PCR tests are now negative for the cohort.

The size of the cohort was 22.3 million unique patients, which the unique combinations can be observed in Table 1. We examined multiple scenarios of interest including patients with more than 1 test, patients with more than 2 tests, patients with two tests within 24 h, patients with a test sequence that was positive ≥ negative ≥ positive, patients with a test sequence that was negative ≥ positive ≥ negative, and patients with >115 days where they are still positive.

**Table 1:** General demographics of testing results for SARS-CoV-2 RT-PCR testing.

	**All patients**	**Patients >1 test**	**Patients >2 tests**	**Two tests within 24 h**	**First test pos, second test neg, third test pos**	**First test neg, second test pos, third test neg**	**>115 days after first positive**
Unique patients	22,203,544	3,867,016	1,199,458	93,735	2,366	37,590	33,443
Positive tests	2,740,271	840,808	302,815	23,669	5,138	38,081	434
Negative tests	27,110,144	10,705,867	5,928,884	226,899	5,175	92,146	64,324
First test positive patients	2,272,133	372,670	88,037	10,107	2,366	–	–
Any test positive patients	2,604,847	705,384	220,514	9,751	2,327	37,421	408
Mean age (Female/Male)	42.7/45.5	44.3/47.6	47.7/53.2	39.4/42.5	48.4/55.4	42.0/44.6	51.1/45.3
Female	55%	57%	59%	52%	53%	58%	59.7%

We examine positivity rate decay in Figure 1 with a negative exponential function, which is evidenced by the positivity rate expressed on the logarithmic scale on the right. At day 10, the positivity rate is about 30%. The decay rate starts at 75% (not 100%), as there is an intrinsic state change in up to 25% of the cohort population.

This retrospective analysis used North Carolina as a reference baseline, as Labcorp has a traditionally high coverage rate of diagnostic testing in the state. TThe 24 h re-test negativity rate (rate of negativity 24 h after Labcorp's first positive PCR test) is 15% in North Carolina, compared to 25% on the national cohort. The decay for both the NC baseline and the national cohort follows a well-defined negative exponential decay rate, which is what is typically expected for this type of viral infection status (15). The uncertainty at >60 days post first-PCR positive test is related to the decreasing number of people getting PCR tested >60 days between follow-up PCR tests.

### False Positive Rates Across US and Within a Control State

This study is an epidemiological study, so the evidence here is not related to any of the quantitative results from PCR testing such as cycle threshold (Ct) values. Manufacturers of testing assays have strict standards for how sensitivity and specificity rates are measured. However, the rates of positivity and negativity in repeat testing can be used to infer various false positive and false negative rates.

**Figure 2:**
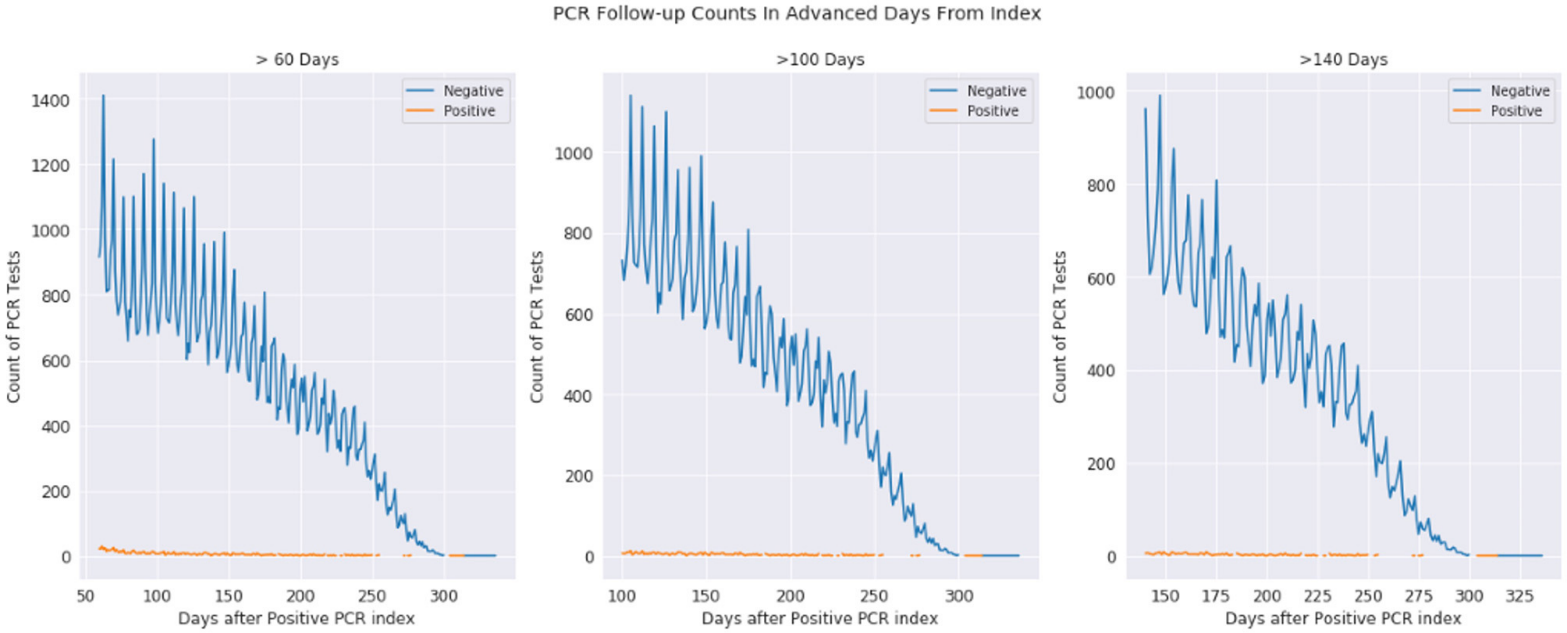
Positive and negative PCR tests 60-330, 100-330, and 140-330 days after first positive PCR test. The plots are the same underlying data, just shown with different time scales. The orange represents positive tests while the blue represents negative tests, where day 0 is a patients first positive PCR test.

In Figure 2, we examine the rate of positive and negative PCR tests from 60 to 350 days after a patient's first positive PCR. The first observation is that we see miniscule rates of positive tests at 100 days and continuing outward. Given these are patients who were once PCR positive at day 0, we observe most who are repeat tested are now negative. The dwindling slope observed is that of less of the population being tested the further observed out from day 0.

The observations from Figures 1 and 2 (as well as information in the Supplemental Material) demonstrate that while infection is active, PCR testing may result in sudden state changes (for example positive–negative–positive) during the active duration. However, for patients who have known to have a positive PCR test on day 0, the rate of positive re-tests is under 1% for any given day 100+ days past the infection. We would expect to see a significant positivity rate for these patients if PCR testing had a significant false positive rate.

### Repeat PCR Testing Within 96 h

There is a sufficient number of patients (3.8 million patients with >1 test) who get repeat testing to draw inferences on effectiveness of testing and uncertainty within repeat testing. We cataloged a series of scenarios where patients take at least three tests, all within a given timeframe. There are three potential outcomes: positive, negative, and “other,” where other can represent various issues from collection to specimen quality.

We first restrict the PCR testing lifecycle to within 96 h between tests. This mean that repeat testing can be at most 8 days, but as soon as 2 days for all three tests. The conditional probabilities of transitioning from one test outcome to the next is represented in the Supplementary Material. For example, for a randomly chosen sample in the cohort, there is 9.2% probability that the first PCR test will be positive. Given that a patient test is positive, we then examine the patient trajectory within the next 96 h. We observe that 40.2% of patients will test negative given that their first test was positive within a 96 h period. Similarly, when a patient test is negative, 96% of the time they do not test positive if get tested again within 96 h. Furthermore, we observe that when the first two tests are negative, the third test is positive 2.4% of the time. When a patient has tested positive followed by a negative test, 17.7% of the time the third test will come back positive within the 96 h time span.

Finally, when a patient has “other” as a test result two times in a row, 85.6% of the third test results will continue to come back as “other”. Similarly, we observe that 2.8% of tests will be positive after two “other” tests. A positive test follows two negative tests for 2.4% of patients.

## Discussion

### False Positive Rates and Long-Term Infections

There is discussion of cycle times related to PCR testing affecting the outcomes of positive PCR tests (as referenced in the introduction) and leading to potential false positives. In our analysis, we were able to index positively infected patients (at day 0) and examine their trajectories at 100+ days after infection. These are patients who technically had two outcomes: either a false positive or a true positive. If the false positive rate is uniformly distributed among all tests, we would expect to see one of three outcomes at 100+ days after infection: the patient is still shedding viral load, the patient is reinfected, or we are observing the natural false positive rate. The rates of positive tests at >115 days is 0.015% (408 patients). There is no evidence of widespread false-positive rates for known positively tested patients at 115+ days.

The long-term infection rate of individuals is not well known because of how many tests are needed to make statistical inferences and the lack of evidence. In our analysis, we rarely see positives at >115 days. While it is possible that a patient could become infected at >180 days, we observe just 4 patients with PCR tests positive >115 days. It is impossible to conclude, but these could be reinfections, long-hauler shedders, or false positives. If we assumed the worst-case scenario that they are reinfections, the positivity rate <0.02% for >115 days would account for the combination of those 3 scenarios.

### Repeat Testing Within 96 h

We used the outcomes of repeat testing results to compare uncertainty in epidemiological characteristics of the Labcorp-tested population. It's important to remember that large reference laboratories like Labcorp likely test a wide range of circumstances, from routine drive-thru testing, at-home testing to critical care settings. Some patients who are tested may be exceptionally symptomatic while others may be “exposed” with no symptoms and are getting a test at the direction of public health officials or to return to work or school. We are surprised to observe that a patient's first Labcorp-documented PCR test for a patient will be followed by a negative PCR test roughly 25% of the time (although the state of North Carolina, where LabCorp has a large market share, is just 15%).

Most likely the main difference is that people simply have true patho-physiological differences of viral load in respiratory mucosa, whereby results can sometimes be negative or positive (or even so close to the line of detection that a patient can test positive and negative within a 24 h period).

It's unlikely the case that the first test was a false positive, based on the evidence presented in the long-term positivity rates for repeat testers and the previous research presented in the introduction, as well as the published EUA approval metrics for positive test detection. We see miniscule rates of positive tests for patients who we know have tested positive for SARS-CoV-2 up to 100 days prior (it's worth noting that it may be possible that the patient was on the tail-end of infection or had a small viral load).

We also considered the case when a patient goes positive, then negative, and then positive again. Given that a patient is positive and then negative within the first 96 h, 17.6% of those patients will test positive on a third test. In some relaxing of COVID-19 protocols, there are some that say that the first subsequent negative test is a pass back into society. The middle test could be a false negative, or it could be a physiological response the body makes in response to complicated dynamics of the immune response. There is evidence of people becoming symptomatic, starting to recover, and then diverging quickly into a state of serious symptoms (16). A negative test after infection carries with it a 17.6% burden that a patient will subsequently test positive, albeit the mechanism of a secondary positive is unknown. We believe there are many other interesting observations from viewing the conditional probability figure and invite the reader to make their own observations.

### Limitations

With this analysis, there are a few caveats that need to be considered. First, this does not account for people who received rapid tests, nor those that may have been tested at an internal lab in an acute care setting. Further, it is important to note that Labcorp has contracts with various government and industrial partners to conduct repeated surveillance of targeted populations (to reiterate, we are using repeat testing as a method to examine uncertainty in the overall population, and are not looking to specifically make inferences about repeat-testing outcomes). Additionally, Labcorp has a self-administered at-home testing kit that was Emergency Use Authorization (EUA) approved for the duration of this cohort study [Pixel by LabCorp^TM^ (17, 18)]. Secondary analysis showed that the number of patients who tested positive using a Pixel test within 96 h of a non-Pixel positive PCR test did not differ significantly from those who tested positive in non-Pixel follow-up tests (Fisher exact, *p* = 0.200). Method of collection was not available in this de-identified dataset, but we do suggest that a good follow-up investigation would be to repeat the analysis to look at changes when sample type is changed between OP and NP. Patients could have been tested through various channels through different locations or facilities.

The biggest caveat remains that it is impossible for us to know in this data where a patient has tested with regards to onset of symptoms. It is possible that a patient's first PCR test was not performed by Labcorp, and thus we would have missing data because their first PCR test was not recorded in the cohort. If this is a significant number of tests, we must be cautious of the metrics reported around second test positivity. We conducted the North Carolina baseline test to get a closer approximation to onset of symptoms at day 0. It's also possible people may be symptomatic and may not get tested for extended periods, which would not be detectable in this analysis. Disagreement between kits is certainly possible and must be mentioned as a potential, albeit slight, possibility.

## Conclusion

Repeat testing of PCR patients presents difficult decisions for clinicians and practitioners. There is little to no evidence of widespread continued infection or reinfection in our testing populations after 115+ days from a patient's first PCR test. Given that a patient test is positive and then negative within 4 days, 17.6% of patients will test positive again if tested within the next 4 days. Further, 25% of patients nationwide (15% within the state of North Carolina) will test negative *just the next day* after testing positive. Even given the sudden change in dynamics of the virus as it takes its course, there is little evidence of a significant false positive rate because of our observations of patients who we know to be PCR positive.

## Data Availability Statement

The datasets generated for this article are not readily available because this data, while de-identified, was generated from a PHI dataset and under HIPAA cannot be publicly posted. Requests to access the datasets should be directed to sullia6@labcorp.com.

## Ethics Statement

Use of the data within Labcorp's COVID-19 registry was approved with waiver of authorization for the use and disclosure of protected health information by Western IRB on March 26, 2020.

## Author Contributions

AS and DAl did the analysis, wrote the draft, and created the hypotheses. DAd, JW, BP, and SL contributed to design, interpretation, discussion, and conclusions. All authors contributed to the article and approved the submitted version.

## Conflict of Interest

The authors declare that the research was conducted in the absence of any commercial or financial relationships that could be construed as a potential conflict of interest. All authors are employees of Labcorp.

## Supplementary Material

Supplementary Figure 1 Conditional Probabilities of repeat PCR testing and have a caption/description of Conditional probabilities of positive (+), negative (−) and other (specimen issues, broken collection containers, etc.). There were 19,719 patients used to construct this cohort analysis. This conditional probability tree allows you to traverse the state changes of testing results from a patients' first, second, and third test.

## References

[1] Mcnaughton CD Adams NM Johnson CH Ward MJ Lasko TA. Diurnal variation in SARS-CoV-2 PCR test results: test accuracy may vary by time of day. medRxiv. (2021). 10.1101/2021.03.12.21253015 PMC8599649 34696614

[2] Harvey RA Rassen JA Kabelac CA Turenne W Leonard S Klesh R. Association of SARS-CoV-2 seropositive antibody test with risk of future infection. JAMA Intern Med. (2021) 181:672–9. 10.1001/jamainternmed.2021.0366 33625463 PMC7905701

[3] Gupta-Wright A MacLeod CK Barrett J Filson SA Corrah T Parris V. False-negative RT-PCR for COVID-19 and a diagnostic risk score: a retrospective cohort study among patients admitted to hospital. BMJ Open. (2021) 11:e047110. 10.1136/bmjopen-2020-047110 33563629 PMC7874904

[4] Garg A Ghoshal U Patel SS Singh DV Arya AK Vasanth S. Evaluation of seven commercial RT-PCR kits for COVID-19 testing in pooled clinical specimens. J Med Virol. (2021) 93:2281–6. 10.1002/jmv.26691 33230819 PMC7753435

[5] Feng H Liu Y Lv M Zhong J. A case report of COVID-19 with false negative RT-PCR test: necessity of chest CT. Jpn J Radiol. (2020) 38:409–10. 10.1007/s11604-020-00967-9 32266524 PMC7136155

[6] Ren X Liu Y Chen H Liu W Guo Z Zhang Y. Application and optimization of RT-PCR in diagnosis of SARS-CoV-2 infection. SSRN Electron J. (2020). 10.2139/ssrn.3546086. [Epub ahead of print].

[7] Arevalo-Rodriguez I Buitrago-Garcia D Simancas-Racines D Zambrano-Achig P Campo R Del Ciapponi A. False-negative results of initial RT-PCR assays for COVID-19: a systematic review. PLoS ONE. (2020) 15:e0242958. 10.1371/journal.pone.0242958 33301459 PMC7728293

[8] Katz AP Civantos FJ Sargi Z Leibowitz JM Nicolli EA Weed D. False-positive reverse transcriptase polymerase chain reaction screening for SARS-CoV-2 in the setting of urgent head and neck surgery and otolaryngologic emergencies during the pandemic: clinical implications. Head Neck. (2020) 42:1621–8. 10.1002/hed.26317 32530131 PMC7307014

[9] Fisher B Seese L Sultan I Kilic A. The importance of repeat testing in detecting coronavirus disease 2019 (COVID-19) in a coronary artery bypass grafting patient. J Card Surg. (2020) 35:1342–4. 10.1111/jocs.14604 32400044 PMC7272872

[10] Lagier JC Colson P Tissot Dupont H Salomon J Doudier B Aubry C. Testing the repatriated for SARS-Cov2: should laboratory-based quarantine replace traditional quarantine? Travel Med Infect Dis. (2020) 34:101624. 10.1016/j.tmaid.2020.101624 32179125 PMC7102645

[11] Li Y Yao L Li J Chen L Song Y Cai Z. Stability issues of RT-PCR testing of SARS-CoV-2 for hospitalized patients clinically diagnosed with COVID-19. J Med Virol. (2020) 92:903–8. 10.1002/jmv.25786 32219885 PMC7228231

[12] Woloshin S Patel N Kesselheim AS. False negative tests for SARS-CoV-2 infection — challenges and implications. N Engl J Med. (2020) 383:e38. 10.1056/NEJMp2015897 32502334

[13] Tang YW Schmitz JE Persing DH Stratton CW. Laboratory diagnosis of COVID-19: current issues and challenges. J Clin Microbiol. (2020) 58:e00512–20. 10.1128/JCM.00512-20 32245835 PMC7269383

[14] Surkova E Nikolayevskyy V Drobniewski F. False-positive COVID-19 results: hidden problems and costs. Lancet Respir Med. (2020) 8:1167–8. 10.1016/S2213-2600(20)30453-7 33007240 PMC7524437

[15] Smith AM Adler FR Perelson AS. An accurate two-phase approximate solution to an acute viral infection model. J Math Biol. (2010) 60:711–26. 10.1007/s00285-009-0281-8 19633852 PMC2841722

[16] Berlin DA Gulick RM Martinez FJ. Severe Covid-19. N Engl J Med. (2020) 383:2451–60. 10.1056/NEJMcp2009575 32412710

[17] LabCorp Receives FDA Authorization to Make At-Home COVID-19 Collection Kits Available Through Retail. Burlington, NC (2020). Available online at: https://www.labcorp.com/coronavirus-disease-covid-19/news/labcorp-receives-fda-authorization-make-home-covid-19-collection-kits-available-through-retail (accessed December 16, 2020).

[18] FDA. FAQ's on Testing for SARS-CoV-2. Available online at: https://www.fda.gov/medical-devices/coronavirus-covid-19-and-medical-devices/faqs-testing-sars-cov-2

