# Supplementary figures and images for: Follow-Up SARS-CoV-2 PCR Testing Outcomes From a Large Reference Lab in the US
Source: Front Public Health. 2021 May 31;9:679012. doi: 10.3389/fpubh.2021.679012 (PMC8200821; doi:10.3389/fpubh.2021.679012)

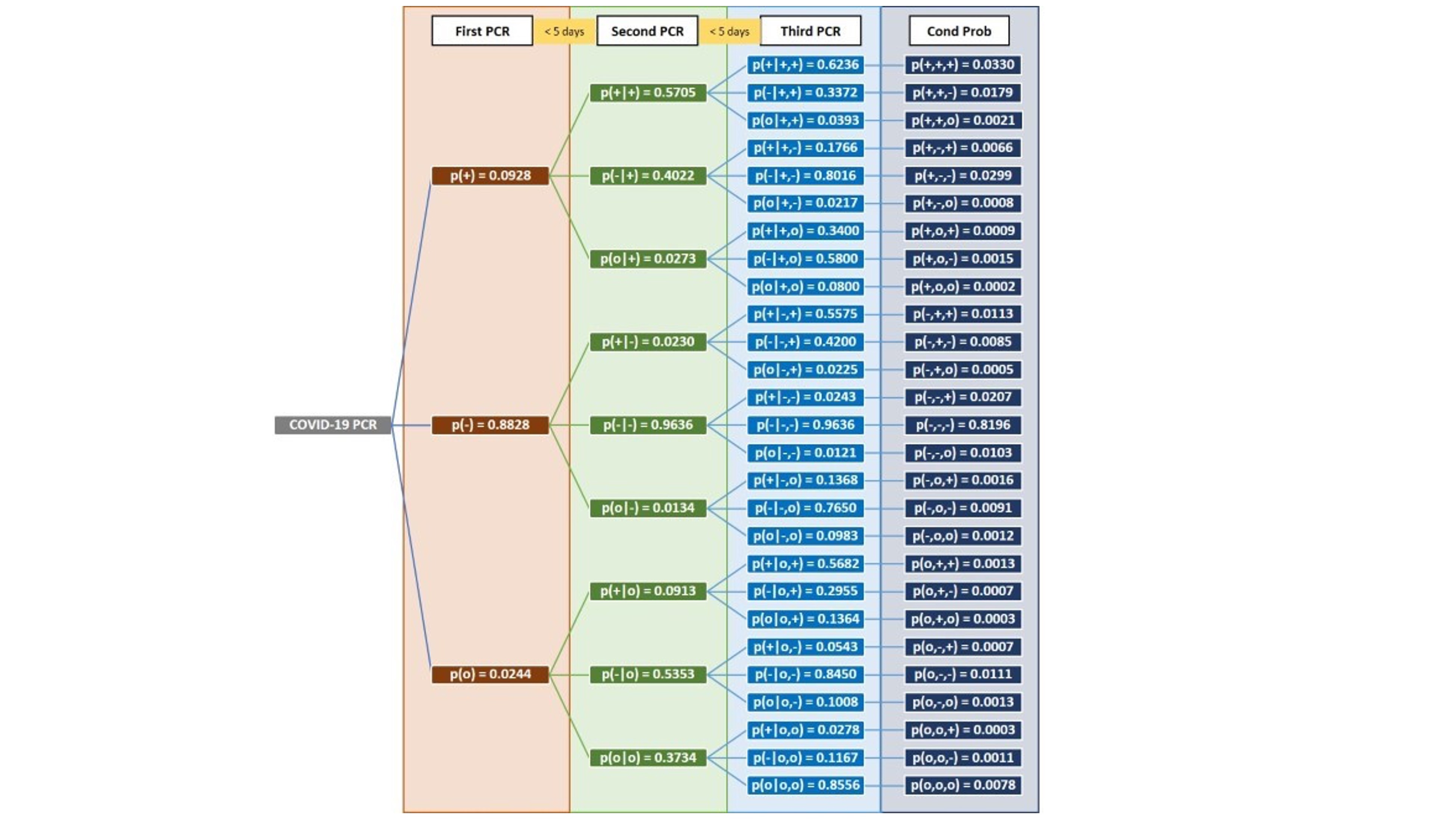

Supplement: Supplementary Figure 1 — Conditional Probabilities of repeat PCR testing and have a caption/description of Conditional probabilities of positive (+), negative (−) and other (specimen issues, broken collection containers, etc.). There were 19,719 patients used to construct this cohort analysis. This conditional probability tree allows you to traverse the state changes of testing results from a patients' first, second, and third test. [file Image_1.JPEG]
